# Ampeliscidae (Crustacea, Amphipoda) from the IceAGE expeditions

**DOI:** 10.3897/zookeys.731.19948

**Published:** 2018-01-23

**Authors:** Rachael A. Peart

**Affiliations:** 1 Coasts and Oceans, National Institute of Water and Atmospheric Research, Wellington, New Zealand

**Keywords:** Ampelisca, Byblisoides, distributions, Haploops, Iceland, key, new species

## Abstract

Ampeliscidae has been recorded extensively from Icelandic waters by many detailed reports. Material collected from the IceAGE (Icelandic marine animals: Genetics and Ecology) 1 and 2 expeditions has resulted in a reasonably expected collection of ampeliscid amphipod species and distributions. However, as seems to be the trend in amphipod systematics, there are ever-present species complexes. Resulting from this, two species new to the genus *Haploops* are presented. Additionally, a new species and new record and key of the genus *Byblisoides* is also presented.

## Introduction

The family Ampeliscidae Krøyer, 1942 is a species diverse group of soft sediment, benthic dwelling amphipods. To date there are 306 described species from only four genera (Horton et al. 2017). Species from this family are known from intertidal to abyssal depths and can range in size from 3 – over 25 mm. Due to living in the first few centimetres of sediment/benthos ampeliscids are important environmental health indicators (accumulation of heavy metals) and vital components of fishery and larger mammal food webs. Some taxa can also form biogenic environments, due to their tube building behavior, providing a habitat for other organisms. Ampeliscid amphipods appear to have a strong depth and sediment delimitation of species (Dauvin et al. 2012), which can help in assessment of geophysical environments.

Ampeliscids have a global distribution, with majority of species recorded from the northern hemisphere. This is mainly due to the extensive combined works from Drs Dauvin, Bellan-Santini and Kaïm-Malka over many years. Whilst these researchers have focused on northern Atlantic waters (along with authors such as Sars 1895, Mills 1967, 1971), there has also been work on various parts of the Pacific Ocean. These include extensive studies on the eastern and Northern Pacific (Barnard 1954, 1961, and 1967, Dickinson 1982, 1983), and the western and tropical Pacific (Gurjanova 1951, 1955, Ren 2006, Dang and Le 2013). There have been very few studies extending into the southern hemisphere.

The scope of this paper is to document the ampeliscid fauna from Icelandic waters collected from the IceAGE 1 and 2 expeditions. This fauna has been widely and elegantly documented (Sars 1895, Bellan-Santini and Dauvin 1988, 1997, 2008, Dauvin and Bellan-Santini 1988, 1990, Dauvin et al. 2012, Kaïm-Malka 2000, 2010, Kaïm-Malka et al. 2016), so there was little expectation of new fauna. Even though previous sampling was extensive and most of the known species were recorded in the current samples, three new species including a new record of the genus *Byblisoides* K.H. Barnard, 1931, not previously known from this region, are documented. Species of Ampeliscidae, like most amphipods, seem to form cryptic species complexes. These new species are often superficially similar morphologically, but as previous researchers on the group have noted the differences are complex but clear, warranting specific status (Kaïm-Malka et al. 2016).

## Materials and methods

The specimens documented in this project are part of the IceAGE project (Icelandic marine Animals: Genetics and Ecology project). The project’s main aim is to combine a variety of areas of biodiversity research and ecological modelling and was managed by the German Centre for Marine Biodiversity Research (**DZMB**). The specimens were collected from two expeditions following a variety of transects in Icelandic waters (see introductory paper, this issue): IceAGE 1 (ME 85-3) on the RV Meteor, sample dates of presented material 28/08/2011 – 22/09/2011; and IceAGE 2 (POS 456) on the RV Poseidon, collection dates of presented material 24/07/2013 – 31/07/2013. All the material processed was collected using Epibenthic Sledge (EBS) and was taken either from the supranet or epinet portion. The material was preserved in 96% ethanol. The amphipods were sorted to family level at two workshops, the first in July, 2016 in Wilhelmshaven, Germany and the second in April, 2017 in Spala, Poland.

Later for species taxonomic examination the material was dissected and examined in glycercol. The pencil drawings of the whole animals were made on dissecting microscope Leica MZ12.5 (with attached camera lucida) and the dissected parts drawn on compound microscope Zeiss Axioskop 2 plus (with camera lucida). Measurements were made dorsally from the tip of the rostrum to the tip of the telson.

Type and other material is deposited in the collections of the Zoological Institut and Museum at the University of Hamburg (**ZMH**) with secondary type material and some material examined deposited in the NIWA Invertebrate Collection (**NIWA**), Wellington, New Zealand.

Abbreviations used include: A – antenna, UL – upper lip, LL – Lower lip, MD – mandible, MX – maxilla, MXP – maxilliped, G – gnathopod, P – pereopod, epim – epimeron, U – uropod, and T – telson.

## Results

**Figure 1.**
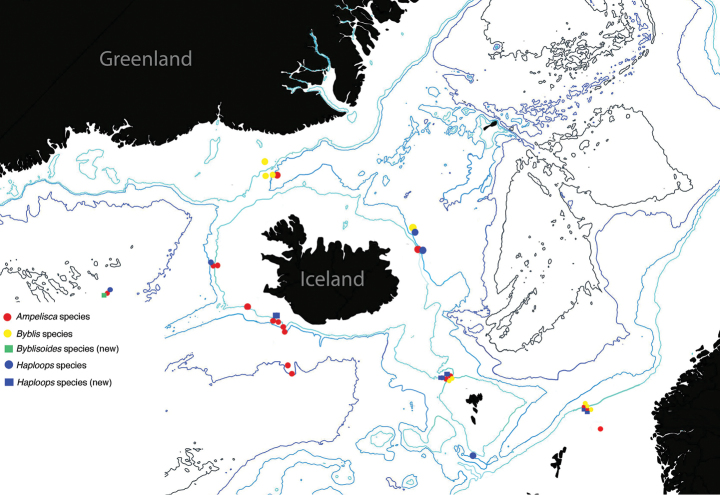
Map to the distribution of species from Icelandic waters in the genera of Ampeliscidae, highlighting the new species documented.

Of the 20 species previously documented from Icelandic waters (Dauvin et al. 2012), this study recorded 13 species (majority in the genus *Ampelisca*) and six species new to Icelandic waters (including three species new to science) (Table 1). Overall, 432 identifiable specimens were examined (there were also a number of damaged specimens that were unidentifiable to species but definitely in the family Ampeliscidae). Of these specimens, 228 belonged to the genus *Ampelisca* (in 9 species), 122 specimens belong to *Haploops* (in 5 species), 81 specimens belong to *Byblis* (in 4 species), and 1 specimen (and species) to *Byblisoides* (Table 1). The distribution of species (Figure 1) shows that as expected *Ampelisca* species occur at the most number of stations and with the greatest depth range.The study had relatively low abundances compared to the BIOICE (Benthic Invertebrates of Icelandic waters) study (Dauvin et al. 2012), with mostly only a few ampeliscids recorded at each station. This is potentially due to differences in sampling gear, depth ranges and number of stations sampled.

**Table 1.** Ampeliscidae amphipods from IceAGE 1 and IceAGE 2 epibenthic sledge collections. (E) represents samples from the epinet bucket of the epibenthic sledge; (EÜ) represents samples from the epinet above the bucket; (S) represents samples from the supranet bucket; and (SÜ) represent samples from the supranet above the bucket.

Species	Station	Locality	Depth (m)	No.	Previous records
*Ampelisca aequicornis* Bruzelius, 1859	IceAGE 1 1033-1(E)	Reykjanes Ridge, South Iceland (shelf)	288.5–293.6	1	Skagerrak (Type), Iceland, Faroe Islands, Bay of Biscay, Norwegian Sea, Arctic North Atlantic, and Northwestern Atlantic (30–983 m)
	IceAGE 2 866-7 (E)	Norwegian Channel	168.8–169.1	4	
	867-1 (E)		290–302.5	49	
**Totals**			**168.8–302.5**	**54**	
*Ampelisca anomala* Sars, 1882 *	IceAGE 2 867-1 (E)	Norwegian Channel	290–302.5	6	Faroes, Bay of Biscay, Norwegian Sea (Type).
**Totals**			**290–302.5**	**6**	
*Ampelisca compacta* Norman, 1882	IceAGE 1 1010-1 (E)	Iceland Basin, South Iceland (slope)	1384.8–1389	15	Iceland, Faroe Islands (268–2082 m)
**Totals**			**1384.8–1389**	**15**	
*Ampelisca gibba* Sars, 1882 *	IceAGE 2				Faroes, Bay of Biscay, Norwegian Seas (type)
	879-5 (E)	Faroe Island Ridge - middle	500.6–510.9	12	
	866-7 (E)	Norwegian Channel	168.8–169.1	16	
**Totals**			**168.8–510.9**	**28**	
*Ampelisca islandica* Bellan-Santini & Dauvin, 1997	IceAGE 1 979-1 (S)	Iceland Basin, South Iceland (deep Sea)	2567.6–2572.2	3	Icelandic waters (type) (884–2082 m)
	983-1 (E)	Iceland Basin, South Iceland (slope)	2568.5–2749.4	8	
	1006-1 (S)1010-1 (E)	Irminger Basin, South Iceland (slope)	1386.8–1390.11384.8–1389	4514	
	1082-1 (S)		704.9–724.4	12	
**Totals**			**704.9–2749.4**	**82**	
*Ampelisca macrocephala* Liljeborg, 1852	IceAGE 2				Iceland, Faroe Islands, Norwegian Seas, Arctic north Atlantic, northwest Atlantic (10–797 m)
	868-3 (S)	Norwegian Channel	587.4–614.4	1	
	879-5 (E)	Faroe Island Ridge - middle	500.6–510.9	8	
**Totals**			**500.6–614.4**	**9**	
*Ampelisca odontoplax* Sars, 1895	IceAGE 1 1017-1 (E)	Iceland Basin, South Iceland (shelf)	891.7–910.3	28	Iceland, Faroe Islands, Norwegian Seas (type), Arctic north Atlantic (139–1993 m)
	1033-1 (E)	Reykjanes Ridges, South Iceland (shelf)	288.5–293.6	2	
	1086-1 (E)	Irminger Basin, South Iceland (slope)	678.5–698.1	2	
	1219-1 (E)	Norwegian Sea, East Iceland	579.1–622.4	2	
	IceAGE 2 867-1 (E) 880-2 (E)	Norwegian ChannelFaroe Island Ridge - middle	290–302.5686–687.4	12	
**Totals**			**288.5–910.3**	**37**	
*Ampelisca uncinata* Chevreux, 1887	IceAGE 1 983-1 (E)	Iceland Basin, South Iceland (Deep Sea)	2568.5–2749.4	2	Iceland, Faroe Islands, Bay of Biscay, Northwestern Atlantic (130–2082 m)
	1006-1(S)	Iceland Basin, South Iceland (slope)	1386.8–1390.1	16	
	1010-1 (E)	Iceland Basin, South Iceland (shelf)	1384.8–1389	24	
	1017-1 (E)		891.7–910.3	3	
	1019-1 (S)	Irminger Basin, South Iceland (Deep Sea)	905.9–913.6	24	
	1054-1 (S)		2537.3–2538.1	3	
	1082-1 (S)	Irminger Basin, South Iceland (Slope)	704.9–724.4	2	
	1086-1 (E)	Denmark Strait, East Greenland	678.5–698.1	7	
	1123-1 (E)		716.5–726	1	
	IceAGE 2867-1 (E)	Norwegian Channel	290–302.5	7	
**Totals**			**290–2749.4**	**89**	
*Ampelisca eschrichtii* Krøyer, 1842	IceAGE 2 879-5 (E)880-2 (E)	Faroe Island Ridge - middleFaroe Island Ridge - middle	500.6–510.9686–687.4	35	
**Totals**			**500.6–687.4**	**8**	
*Byblis crassicornis* Metzger, 1875	IceAGE 1 1194-1 (S)	Norwegian Sea, north-east Iceland	1573.5–1579.5	3	Iceland, Faroe Islands, Norwegian Seas, Arctic north Atlantic, northwestern Atlantic (180-2082)
	IceAGE 2	Norwegian Channel			
	867-1 (E)		290–302.5	1	
	868-3 (E)		587.4–614.4	15	
	869-3 (E)	Faroe Island Ridge - middle	846.4–868.4	1	
	879-5 (E)		500.6–510.9	3	
**Totals**			**290–1579.5**	**23**	
*Byblis erythrops* Sars, 1882 *	IceAGE 2 867-1 (E)	Norwegian Channel	290–302.5	11	Faroe Island, Norwegian Seas, Arctic Atlantic, northwestern Atlantic
**Totals**			**290–302.5**	**11**	
*Byblis medialis* Mills, 1971	IceAGE 2				Iceland, Northwest Atlantic (type), (535–2100 m)
	868-3 (EÜ)	Norwegian Channel	587.4–614.4	1	
	880-2 (E)	Faroe Island Ridge - middle	686–687.6	18	
**Totals**			**587.4–687.6**	**19**	
*Byblis minuticornis* Sars, 1879	IceAGE 1 1119-1 (S)	Denmark Strait, East Greenland	696.9–706.4	2	Iceland, Faroe Islands, Bay of Biscay, Norwegian Seas (type), Arctic Atlantic (69–1910 m)
	1123-1 (E)		716.5–726	2	
	1132-1 (SÜ)		316.5–318.1	1	
	IceAGE 2	Norwegian Channel			
	868-3 (E)		587.4–614.4	1	
	869-3 (E)	Faroe Island ridge - middle	846.4–868.4	5	
	879-5 (E)		500.9–510.9	3	
	880-2 (E)		686–687.4	14	
**Totals**			**316.5–868.4**	**28**	
*Byblisoides bellansantini* sp. n.*	IceAGE 1 1054-1 (S)	Irminger Basin, South Iceland (deep sea)	2537.3–2538.1	1	
**Totals**			**2537.3–2538.1**	**1**	
*Haploops carinata* Liljeborg, 1855	IceAGE 1 1057-1 (E)	Irminger Basin, South Iceland	2504.7–2531.8	1	Iceland, Faroe Islands, Norwegian Seas (types) (264–1750 m)
**Totals**			**2504.7–2531.8**	**1**	
*Haploops islandica* Kaim-Malka, Bellan-Santini & Dauvin, 2016	IceAGE 1 1082-1 (S)	Irminger Basin, South Iceland	704.9–724.4	1	Iceland (Type), Faroe Islands (283–1727 m)
	1194-1 (S)	Norwegian Sea, north-east Iceland	1573.5–1579.5	2	
	1219-1 (E)	Norwegian Sea, east Iceland	579.1–622.4	1	
	IceAGE 2	Norwegian Channel			
	867-1 (E)	South-East Faroer Channel	290–302.5	12	
	868-3 (E)		587.4–614.4	7	
	876-5 (E)		554.3–674.8	2	
**Totals**			**290–1579.5**	**25**	
*Haploops tubicola* Liljeborg, 1855	IceAGE 1 1219-1 (E)	Norwegian Sea, east Iceland	579.1–622.4	1	Iceland, Faroe Islands, Norwegian Seas (type), Bay of Biscay, Arctic Atlantic, northwestern Atlantic (10–2886 m)
**Totals**			**579.1–622.4**	**1**	
*Haploops dauvini* sp. n.*	IceAGE 2 868-3 (S)		587.4–614.4	3	
	879-5 (E)	Norwegian Channel	500.6–510.9	8	
	880-2 (E)	Faroe Island Ridge - middle	686–687.4	1	
**Totals**			**500.6–687.4**	**12**	
*Haploops kaimmalkai* sp. n.*	IceAGE 1 1010-1 (E)	Iceland Basin, South Iceland (slope)	1384.8–1389	1	
	IceAGE 2	Faroe Island Ridge - middle Norwegian channel			
	880-2 (E)		686–687.4	3	
	868-3 (E)		587.4–614.4	79	
**Totals**			**587.4–1389**	**83**	

*species not previously recorded in Icelandic waters

## Systematics

### Ampeliscidae Krøyer, 1842

#### Byblisoides

Taxon classificationAnimaliaAmphipodaAmpeliscidae

K.H. Barnard, 1931

##### Diagnosis

(adapted from Barnard and Karaman 1991). Antenna 1–2 flagella with less than 5 articles. Maxilliped palp article 3 not produced. Pereopod 7 basis posterior margin angled and expanding ventrally. Telson longer than broad and cleft more than half of length. Urosomite 1 produced to form large unilobed carina.

#### Byblisoides bellansantiniae sp. n.

Taxon classificationAnimaliaAmphipodaAmpeliscidae

http://zoobank.org/ABDDCB9B-9C4E-4BE2-A986-9BC876C2465B

Figs 2 , 3 , 4

##### Type material.

Holotype, female, 14 mm, ZMH K-47035, station IceAGE 1, 1054-1, Irmiger Basin, South Iceland, 031°22.60'W – 031°22.18'W, 61°36.19'N – 61°36.97'N, 2537.3 – 2538.1 m, ME85 - 3, supranet bucket of EBS, 07.09.2011.

##### Type locality.

Irminger Basin, South Iceland.

##### Etymology.

Named for Dr Denise Bellan-Santini, whose extensive research on the Ampeliscidae has greatly aided this study.

##### Diagnosis.

Anteroventral margin of head rounded and produced to length of anterior margin of head. Antenna 1 short, reaching to three-quarters of antenna 2 peduncle article 4. Pereopod 7 carpus anterior margin without plumose setae. Pereopod 7 basis posteroventral corner rounded. Uropod 2 inner ramus bearing marginal robust setae.Description. Based on holotype female 14 mm length. Head anteroventral corner produced forward, reaching almost level with the anterodorsal corner, anterior margin, excavate at antenna 2 insertion, antennal lobe concave, with two acute points, rostrum absent, head longer than deep, ventral margin slightly sinuous. Antenna 1 short, reaching to three-quarters length of antenna 2 peduncle article 4; peduncle article 1 subequal in length to article 2 (1.1 ×), article 2 longer than article 3 (3 ×), article 3 shorter than article 1 (0.3 ×); flagellum shorter than peduncle, comprising of three articles (article 1 longest), ventral margin of both peduncle and flagellum with long plumose, slender setae. Antenna 2 comparatively stout, reaching to just under half of the body length; peduncular article 4 subequal in length to article 5, article 5 ventral margin slightly serrate, with long slender setae; flagellum shorter than peduncle, with 4 articles.

Mandible molar well-developed and triturating, seven plumose robust setae in accessory setal row; incisor toothed; lacinia moblis with many teeth of different sizes; palp long, article 1 very short, article 2 longer than article 3 (2.2 ×) and curved with sparse setae, article 3 longer than article 1 (3.2 ×), moderately setose. Lower lip two lobed, inner plate half height of outer. Maxilla 1 inner plate rounded and small, covered in setules, no long setae; outer plate topped with toothed robust setae; palp with two articles, article 2 reaching to end of teeth on outer plate, tipped with five long plumose setae and five facial slender setae. Maxilla 2 inner and outer plates of equal height and width, both tipped with long plumose setae. Maxilliped inner plate very short, rounded, tipped with three long robust setae and two slender setae; outer plate twisted around palp, inner lateral margin lined with toothed robust setae becoming slender plumose setae distally; palp broad and subchelate in aspect, article 2 expanded and long bearing many strong slender plumose setae, article 3 narrow and folded over article 2, reaching only a third of the length, strongly setose, article 4 curved and acutely tipped, reaching half the length of article 3.

Gnathopod 1 coxa reaching to edge of anterior margin, coxa expanded distally, ventral margin narrowly curved lined with long plumose slender setae, plus a few medial plumose setae; basis narrow, lateral margins lined with long plumose, slender setae, medial setae long and plumose; merus slightly lobate and strongly setose particularly posteriorly; carpus longer than merus and longer than propodus (1.2 ×), strongly setose, particularly medially and posteriorly with long plumose setae, not lobate; propodus ovoid, weakly subchelate, palm not well defined, anterior and posterior margins lined with long plumose slender setae; dactylus short and curved, a third the length of the propodus, inner margin with a distal tooth and sparse slender setae. Gnathopod 2 coxa similar length to coxa 1, ventral margin curved (unevenly), fringed with medial and marginal sparse long plumose setae; basis long and narrow lateral margins with long slender plumose setae, merus with subacute posterior lobe, long plumose setae on both the anterior and posterior margins; carpus longer than merus and longer than propodus, narrow and not lobate, covered in long, plumose setae; propodus narrow, covered in long plumose setae; dactylus short, about a third the length of the propodus and slightly curved, inner margin setose.

Pereopod 3 coxa similar to coxa 2; basis long and narrow, anterior margin with short sparse setae, posterior margin without setae; merus narrow, shorter than basis, subequal to carpus and propodus together, both margins with sparse long plumose setae; carpus with long plumose setae on posterior margin; propodus longer than carpus, short sparse setae on posterior margin, posterior margin slightly concave; dactylus long and narrow, slightly curved, shorter than propodus. Pereopod 4 coxa subrectangular, posterior margin with extended rounded lobe, posterior margin below lobe concave, ventral margin almost straight and sparsely setose; basis same length as coxa with setose lateral margins; ischium setose along posterior margin; merus long and narrow, shorter than basis, longer than carpus and propodus together, setose with plumose setae along complete length of posterior margin and distal half of anterior margin; carpus shorter than propodus, setose along posterior margin; propodus long and narrow, setose on proximal posterior margin; dactylus long, narrow and straight, shorter than propodus.

Pereopod 5 basis almost rounded narrow distally, anterior margin broadly rounded, lined with long plumose setae, anterior margin bilobed without setae; ischium with acute posterior lobe; merus longer than ischium with only one long plumose seta on anterior margin, no setae on posterior margin; carpus longer than merus, longer than propodus, only setose along anterior margin (all plumose), not lobate; propodus narrow, setose along anterior margin, not lobate; dactylus short, strongly curved and smooth. Pereopod 6 basis nearly circular, subquadrate distally, anterior margin lined with short slender setae, and one long plumose seta, posterior margin without setae; ischium with acute posterior lobe; merus longer than ischium, not lobate, anterior margin with one long slender seta and one long robust seta, posterior margin without setae; carpus longer than merus and longer than propodus, anterior margin lined with eight long robust setae, anterior margin no setae, distal corner with two long robust setae; propodus long and narrow not produced into a distal lobe, anterior margin lined with four short plumose robust setae, distal corner bearing three long strong setae; dactylus short, curved and smooth. Pereopod 7 basis widest distally, ovoid, rounded, medial surface setose, anterior margin with sparse setae, ventral and partly posterior margin lined with long plumose setae all the way to the junction with the ischium, posterior corner slightly serrate; ischium short and not setose, except for anterodistal corner; merus longer than ischium, not lobate, anterior margin with two long robust setae, posterior with three long plumose setae and distally with one long plumose and one robust seta; carpus narrow, longer than merus, subequal to propodus, anterior margin with three robust setae and two distal robust setae, posterior margin without marginal setae, but with four long distal robust setae; propodus long and narrow, anterior margin without setae, posterior margin with two robust setae; dactylus long, straight and narrow, slightly shorter than propodus.

Pleon. Epimeron 1 posteroventrally broadly rounded, no tooth or setae. Epimeron 2 posteroventrally broadly rounded, no tooth or setae. Epimeron 3 posteroventrally produced to form a small subacute tooth, posterior margin straight, no ventral setae. Urosomite 1 produced into a straight dorsally, curved ventrally carina, unilobed when viewed from dorsal. Uropod 1, in situ, reaching to tip of uropod 2 rami; peduncle longer than rami, outer margin lined with three short robust setae, inner margin lined with three proximal, long plumose setae and five long robust setae, row of seven short, robust medial setae; rami subequal in length, outer ramus lined with six robust setae and three slender setae; inner ramus with hair-like setal fringe proximally and distally with four robust marginal setae. Uropod 2 peduncle same length as inner ramus but longer than outer ramus, inner margin with two robust lateral setae and six medial robust setae; outer ramus with four short medial robust setae, one long subterminal robust seta; inner ramus longer than outer ramus with proximal hair-like fringe and two long marginal distal robust setae. Uropod 3 peduncle much shorter than rami, with three distal, medial short robust setae, and two lateral robust setae; inner ramus slightly longer than outer, both rami leaf-shaped (broadest proximally); inner ramus lined with long slender plumose setae and four robust setae laterally (outer margin), five robust setae on inner margin; outer rami inner margin with long slender, plumose setae, outer margin with seven short robust setae laterally. Telson longer than wide (1.65 ×), 78 % cleft; each lobe concavely truncated distally, each lobe with three dorsal robust setae, two slender dorsal setae, one robust apical and one slender apical seta.

Remarks. *Byblisoides* is the smallest of the Ampeliscid genera with only seven species (including this species). However, the species fall into the same traps as the rest of the ampeliscids, in that they have relatively similar morphology needing a combination of characters to provide differences. Usually a species wouldn’t be described from a single specimen, but as this is a new record for the Icelandic region and distinctly a new species it has been done. *Byblisoides bellansantiniae* sp. n. fits the most recent diagnosis of the genus (Barnard and Karaman 1991) in the flagella of antennae 1 – 2 with 4 or fewer articles. Article 3 of maxilliped palp unproduced. Article 2 of pereopod 7 with posterior margin oblique and article expanding ventrally, anterior margin of postero-ventral lobe near junction with article 2 usually setose. Telson much longer than broad, cleft much more than half its length.

This species also aligns to the description of the genus in the same publication, the only slight difference is with the lower part of posterior margin on coxa 4 being angled to the anterior margin (not parallel). *Byblisoides bellansantiniae* sp. n. has the closest affinities to *B. profundi* Mills, 1971 from the Gay Head – Bermuda transect from a depth of 4600–4900 m. The ventral shape of the coxa and general shape of pereopod 7 basis draw these species together. However, they are distinct because of the shape of the anteroventral corner of the head (produced and rounded in *B. bellansantiniae* sp. n. and not produced in *B. profundi*), and setation of basis of pereopod 7 (medially setose in *B. bellansantiniae*, not setose in *B. profundi)*. Other differences include the setation of the carpus and propodus of pereopods 5 and 6 (without setae *B. bellansantiniae*, strong plumose setae *B. profundi*).

A key to the species of *Byblisoides* is provided below. Built on that provided by J.L. Barnard, 1961.

**Figure 2.**
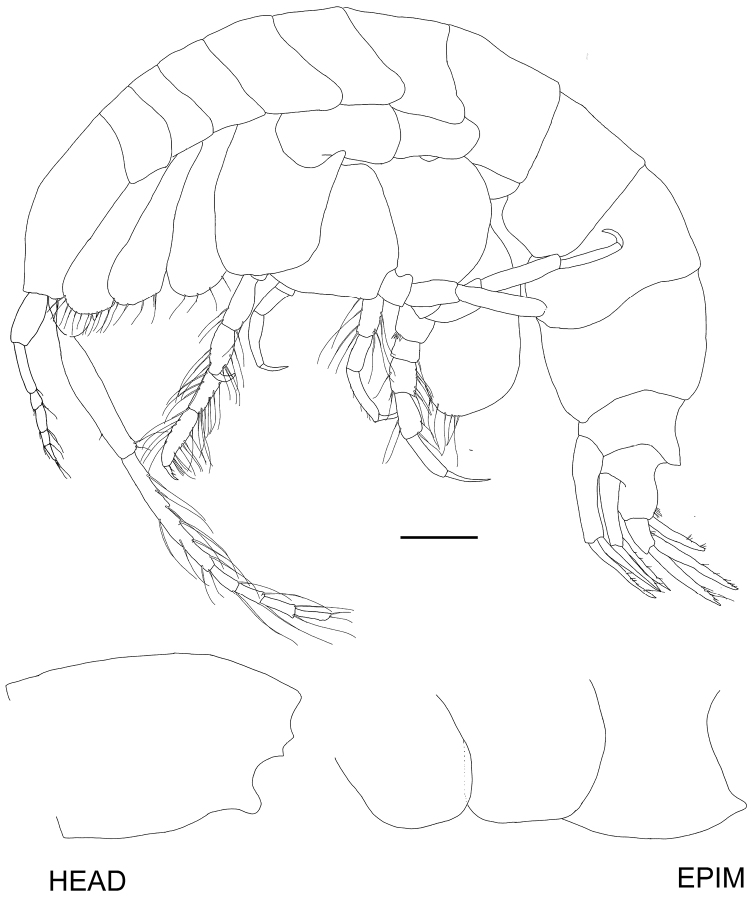
*Byblisoides bellansantiniae* sp. n. Holotype, female, 14 mm, ZMH K-47035, Irminger Basin, Iceland, 2537.3–2538.1 m. Whole animal, head and epimeron. Scale for habitus represents 1 mm.

**Figure 3.**
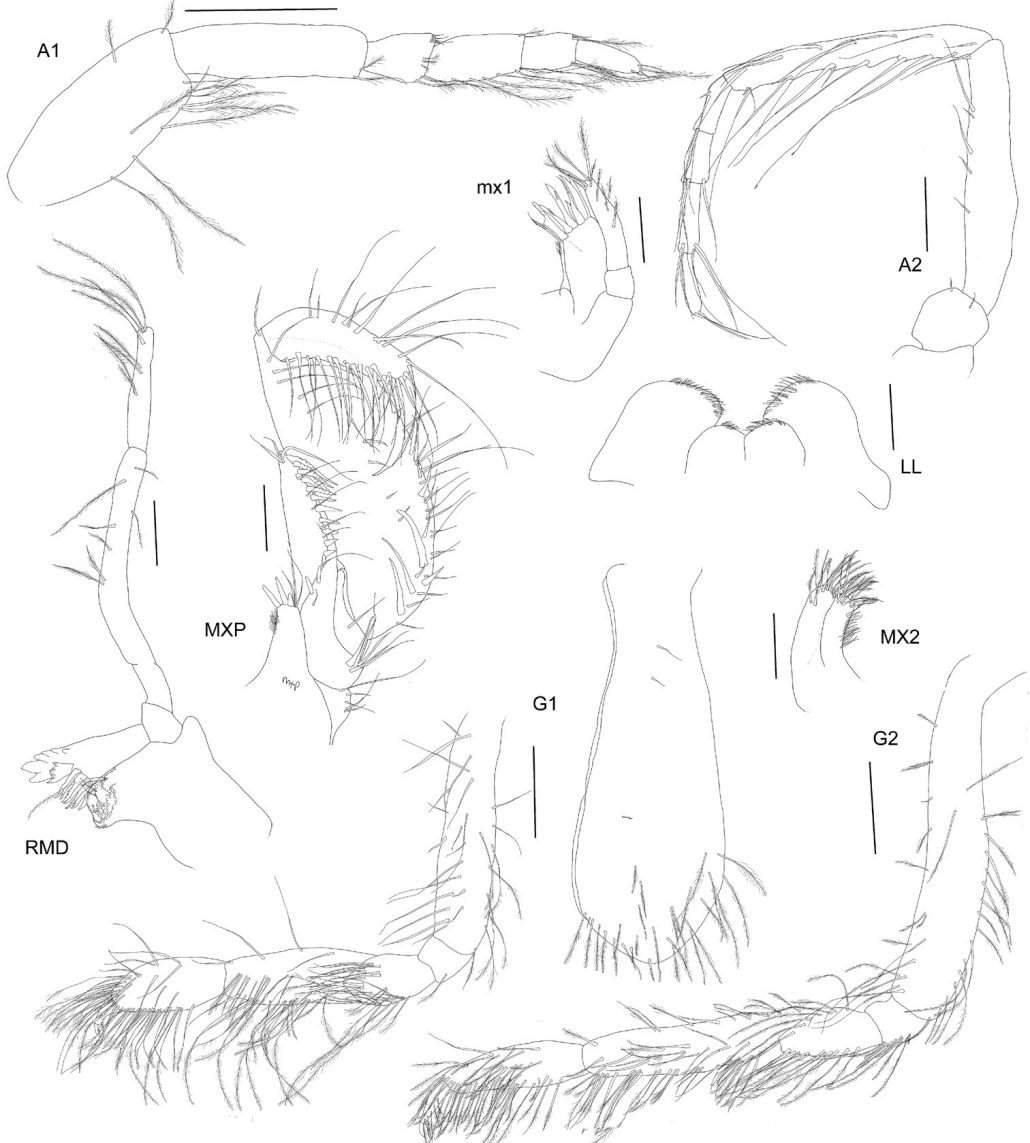
*Byblisoides bellansantiniae* sp. n. Holotype, female, 14 mm, ZMH K-47035, Irminger Basin, Iceland, 2537.3–2538.1 m. Antennae 1–2, gnathopods 1–2 scales represent 0.5 mm. Mouthparts scales represent 0.2 mm.

**Figure 4.**
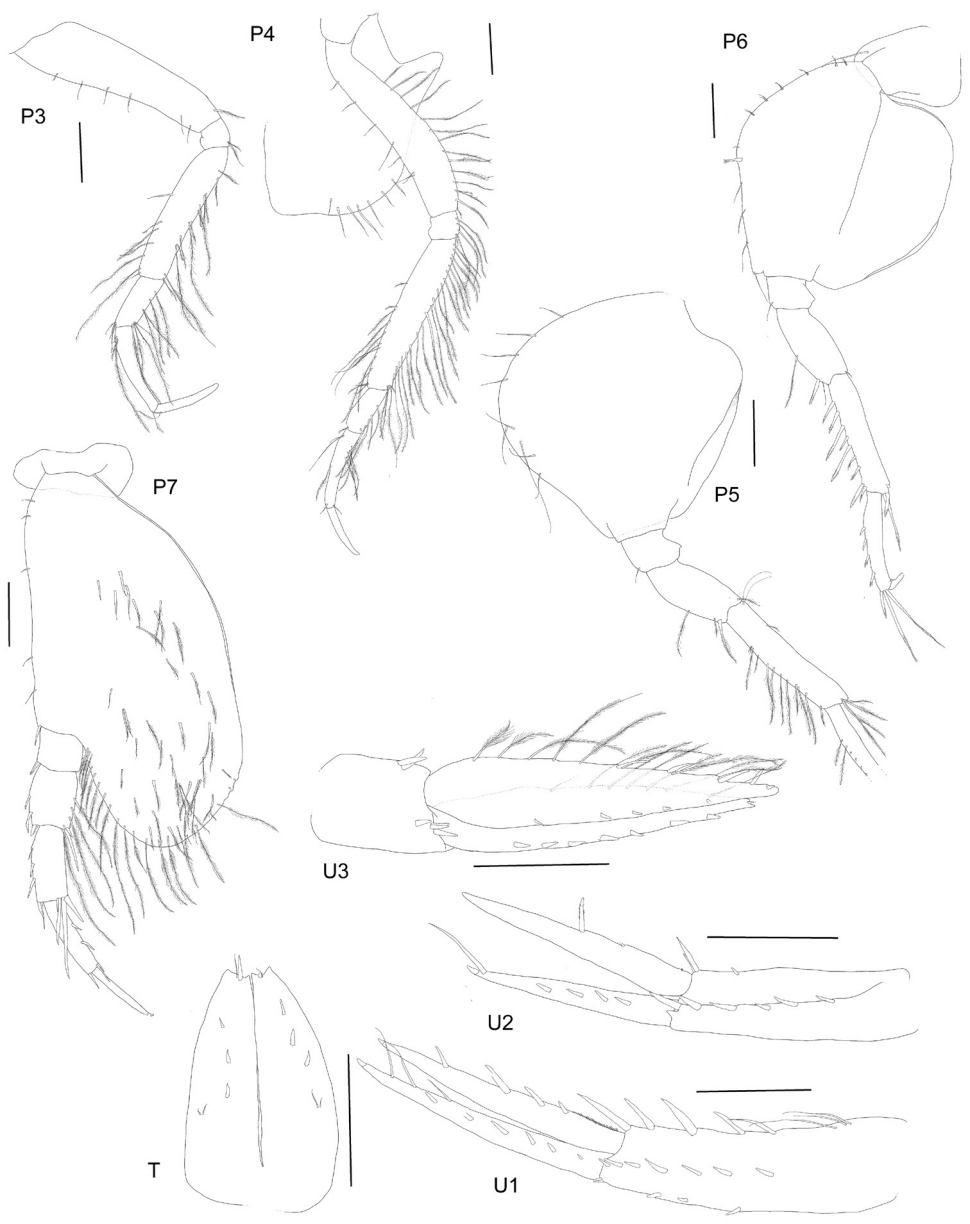
*Byblisoides bellansantiniae* sp. n. Holotype, female, 14 mm, ZMH K-47035, Irminger Basin, Iceland, 2537.3–2538.1 m. Scales represent 0.5 mm

##### Distribution.

Southern Iceland, North Atlantic. Depths 2500–2600 m.

##### Key to species of *Byblisoides*

**Table:** 

1	Anterior edge of carpus of pereopod 7 with four long plumose setae	***B. juxtacornis* K.H. Barnard, 1931**
–	Anterior edge of carpus of pereopod 7 without plumose setae	**2**
2	Urosomite 1 not obviously produced to form a carina (from lateral view)	***B. esferis* J.L. Barnard, 1961**
–	Urosomite 1 obviously produced to form a carina (from lateral view)	**3**
3	Urosomite 1 carina bilobed (from dorsal view)	**4**
–	Urosomite 1 carina not bilobed (from dorsal view	**5**
4	Uropod 2 inner ramus with robust setae	***B. blasensis* J.L. Barnard, 1964**
–	Uropod 2 inner ramus without robust setae	***B. arcillis* J.L. Barnard, 1961**
5	Pereopod 7 basis ventral lobe acute	***B. plumicornis* Ledoyer, 1978**
–	Pereopod 7 basis ventral lobe rounded	**6**
6	Anteroventral corner of head produced and rounded	***B. bellansantiniae* sp. n.**
–	Anteroventral corner of head not produced	***B. profundi* Mills, 1971**

#### Haploops

Taxon classificationAnimaliaAmphipodaAmpeliscidae

Liljeborg, 1856

##### Diagnosis

(adapted from Barnard and Karaman 1991). Antenna 1–2 flagella with more than five articles. Maxilliped palp article 3 expanded and usually produced. Pereopod 7 basis with parallel margins (sometimes straight and sometimes concave), narrow posteroventral lobe present. Telson of varying lengths to widths, usually cleft more than half.

#### Haploops dauvini sp. n.

Taxon classificationAnimaliaAmphipodaAmpeliscidae

http://zoobank.org/B1A77932-F41F-42BE-9D8A-98C6EA8403C7

Figures 5 , 6 , 7

##### Type material.

Holotype, female, 7 mm, ZMH K-47038, station IceAGE 2, 868-3, Norwegian Channel, 000°15.51'E – 000°15.86'E, 62°09.14'N – 62°10.30'N, 587.4 – 614.4 m, POS-456, from the supranet bucket of EBS, 25.07.2013. Paratypes: ZMH K-47039, female 6 mm; NIWA 123641, female, 7 mm, same collection data as holotype.

##### Additional material examined.

ZMH K-47042, ZMH K-47043, 8 specimens, station IceAGE 2, 879-5, Faroe Island Ridge (middle), 008°34.32'W – 008°36.22'W, 63°06.10'N – 63°05.62'N, 500.6 – 510.9 m, POS-456, 31.07.2013. ZMH K-47040, 1 specimen, station IceAGE 2, 880-2, Faroe Island Ridge (middle), 008°09.42'W – 008°11.22'W, 63°23.36'N – 63°24.62'N, 686 – 687.4 m, POS-456, 31.07.2013.

##### Type locality.

Norwegian Channel, North Atlantic Ocean.

##### Etymology.

This species is named for Dr Jean-Claude Dauvin, whose extensive work on the family Ampeliscidae was invaluable for the description of this species.

##### Diagnosis.

Eyes absent. Head anterior margin straight. Antenna 1 article 1 almost half the length of article 2. Antenna 2 peduncular article 4 subequal in length to article 5. Gnathopod 1 carpus subequal in length to the propodus. Gnathopods and pereopods without setal fringe. Pereopod 4 coxa broad. Pereopod 5 basis almost circular. Pereopod 7 basis narrow. Uropod 1 rami subequal in length. Telson with 1 distal robust seta per lobe.

##### Description.

Based on holotype adult female, 7 mm in length.

Both pereon and pleon without dorsal setae. Head almost as deep as long, rostrum absent, anterior margin straight and almost perpendicular to dorsal margin. Corneal lenses absent. Antenna 1 as long as antenna 2, close to half body length; peduncular article 1 shorter than article 2 (0.6 ×), article 2 longer than article 3 (3 ×), article 3 shorter than article 1 (0.5×); flagellum short with 16 articles, fringed ventrally with long, weakly plumose setae. Antenna 2 close to half the body length; peduncular article 4 approximately subequal to article 5 (0.9 ×); flagellum with 15 articles; peduncle and flagellum fringed with long, weakly plumose setae.

Upper lip distally notched, lightly setose. Mandible molar well developed and triturating, with 7 plumose robust setae in the accessory setal row; palp long, article 2 shorter than article 3 (0.85 ×); article 2 weakly setose, article 3 strongly setose with plumose setae. Lower Lip with inner and outer lobe, inner half the height of the outer. Maxilla 1 inner plate with 1 apical strong plumose seta and 4 accessory simple facial setae; palp with two articles, second article reaching past length of outer plate, with 4 robust setae distally and four slender, plumose facial setae. Maxilla 2 plates of similar widths, both with long plumose setae. Maxilliped inner plate elongated with a rounded tip with one short robust seta and nine long plumose slender facial setae; outer plate ovoid reaching to ¾ length of palp article 2, inner lateral margin with robust setae tending to long plumose setae distally; palp article 4 longer than article 3, and inserted subterminally, inner margin setose.

Pereon. Gnathopod 1 coxa roughly teardrop shape, expanded and rounded distally. In situ, reaching to level of anterior margin of head, ventral margin broadly and evenly rounded with fringe of long plumose setae, scattered setae medially; basis shorter than coxa, same length as carpus and propodus together, lateral margins and medially fringed with long plumose setae and occasional short non-plumose setae; merus slightly lobate with long plumose setae on posterior margin; carpus longer than merus, slightly longer than propodus (1.15 ×), with slight rounded posterior lobe, posterior margin bearing many long, plumose slender setae; propodus ovoid, subchelate, posterior margin slightly serrate, strongly setose on both posterior and anterior margin; dactylus long and curved, inner margin setose, reaching to half of length of propodus. Gnathopod 2 coxa 2/3 length of coxa 1, narrowing slightly distally, ventral margin rounded with sparse plumose setae; basis longer than coxa, similar length to carpus and propodus combined, lateral margins with long plumose slender setae; merus non-lobate; carpus longer than merus and longer than propodus (1.6 ×), non-lobate, long plumose setae on both posterior and anterior margins (more dense on posterior margin); propodus ovoid, subchelate, palm not strongly defined, long, plumose setae on both anterior and posterior margins; dactylus shorter than propodus (0.6×), with a setose inner margin.

Pereopod 3 coxa similar in length to coxa 2, ventral margin rounded with sparse long plumose setae; basis longer than coxa, just shorter than merus + carpus + propodus, lateral margins with long, plumose setae; merus shorter than basis, longer than carpus and propodus combined, sparsely setose on both posterior and anterior margins, not inflated; carpus short with long plumose setae on the posterior margin only; propodus longer than carpus, long, plumose setae on anterior margin plus two on the posterior margin, posterior margin straight (not concave or convex); dactylus shorter than carpus and propodus combined (longer than propodus individually), not setose, straight. Pereopod 4 coxa broad with rounded extended posterior lobe, ventral margin rounded (no acute corners) with sparse, plumose setae; basis longer than coxa, fringed laterally with long, plumose setae on both margins, shorter than merus + carpus + propodus; merus narrow, longer than carpus and propodus combined, long plumose setae only at distal end of article; carpus short with posterior, long, plumose setae only; propodus longer than carpus with sparse (2 on anterior and 1 on posterior) plumose setae; dactylus shorter than propodus, straight and without setae.

Pereopod 5 basis broadly rounded, almost circular, anterior margin with a few short setae; distal articles broken off. Pereopod 6 basis almost circular, not as broad as basis 5, anterior margin with sparse small setae, posterior margin rounded; ischium with acute posterior lobe; merus same length as ischium, weakly setose; carpus longer than merus, slightly shorter than propodus (0.85 × not including posterior lobe), anterior margin with two weak robust setae, posterior margin with two rows of stout robust setae (2 and 4), and extended to form a lobe bearing 6 strong, robust setae increasing in length laterally; propodus narrower than carpus, anterior margin with two weak robust setae, posterior margin without robust setae, anterior margin produced slightly to form a weakly setose lobe; dactylus strongly curved and smooth, much shorter than propodus. Pereopod 7 basis moderately narrow (length without lobe /width = 2.0 ×), anterior and posterior margins slightly concave, medial surface with numerous long, plumose setae, posterior-distal lobe narrow, rounded and deflected, reaching to ¾ length of ischium, with a few marginal setae; ischium without anterodistal robust setae; merus subrectangular, longer than ischium (1.8 ×), subequal in length to carpus, anterior margin with one marginal and one distal robust seta, not lobate, posterior margin not lobate with two marginal and one distal robust seta; carpus subovoid, broad (width/length = 0.75×), anterior margin slightly lobate distally, with three marginal robust setae, posterior margin not lobate with two long marginal robust setae and one distal robust seta; propodus narrow, less than half the length of the carpus, no setae; dactylus broken off.

Pleon smooth. Epimeron 1 posteroventral corner broadly rounded, no tooth. Epimeron 2 posteroventral corner broadly rounded, no tooth. Epimeron 3 subquadrate, no tooth and no setae, dorsal margin with sparse, very short setae. Urosomite 1 slightly raised to form a small rounded carina, dorsal margin bearing a few very short setae. Urosomites 2–3 fused.

Uropod 1, in situ, reaching one third of the length of uropod 2 rami; peduncle shorter than rami, outer margin with two robust setae, inner margin with three slender setae, one strong robust seta medio-distally; rami subequal in length, both curved and gently tapering to a subacute tip; outer ramus without setae; inner ramus with two robust setae. Uropod 2 peduncle longer than rami, inner margin with 2 slender setae and two long robust setae distally; rami subequal in length, narrow tapering to a rounded tip; outer ramus with one marginal robust seta; inner ramus with four marginal robust setae. Uropod 3 peduncle shorter than rami (0.65×), and without setae; rami long and narrow, even width along length, not tapering, truncated apically, subequal in length; outer ramus two robust setae distally, long plumose setae on distal half of each margin; inner ramus with two strong robust setae on outer margin and one robust seta distally, plumose setae on distal part of inner margin, hair-like fringe on proximal half of outer margin. Telson slightly longer than wide (1.2 ×), cleft to 68%, each lobe apically rounded subquadrately; each lobe with two slender dorsal setae, one apical slender setae and one apical robust seta.

Male. No males availables in the samples.

**Figure 5.**
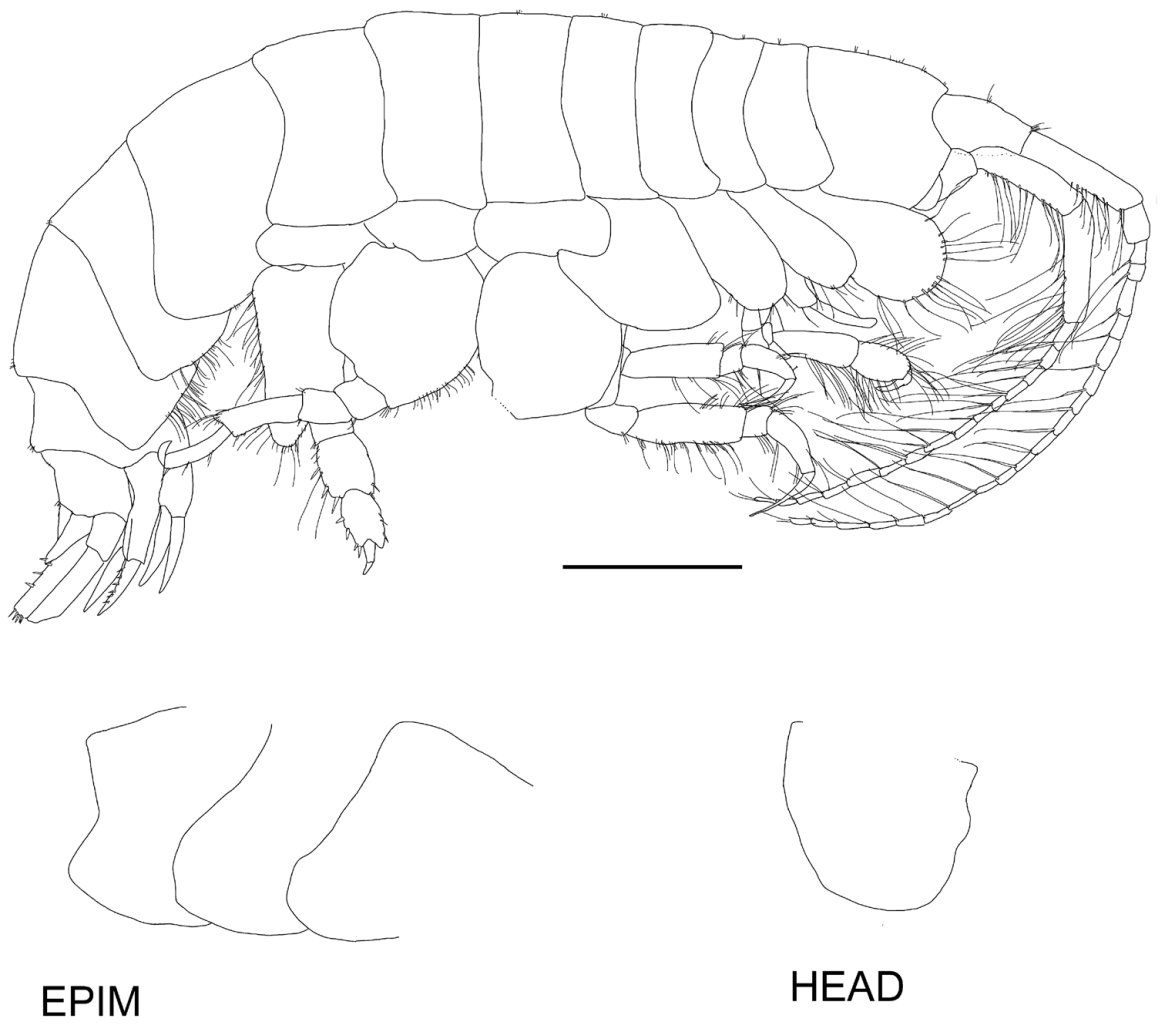
*Haploops dauvini* sp. n., holotype, female, 7 mm, ZMH K-47038, Norwegian Channel, 587.4–614.4 m. Whole animal, head and epimeron. Habitus scale represents 1 mm.

**Figure 6.**
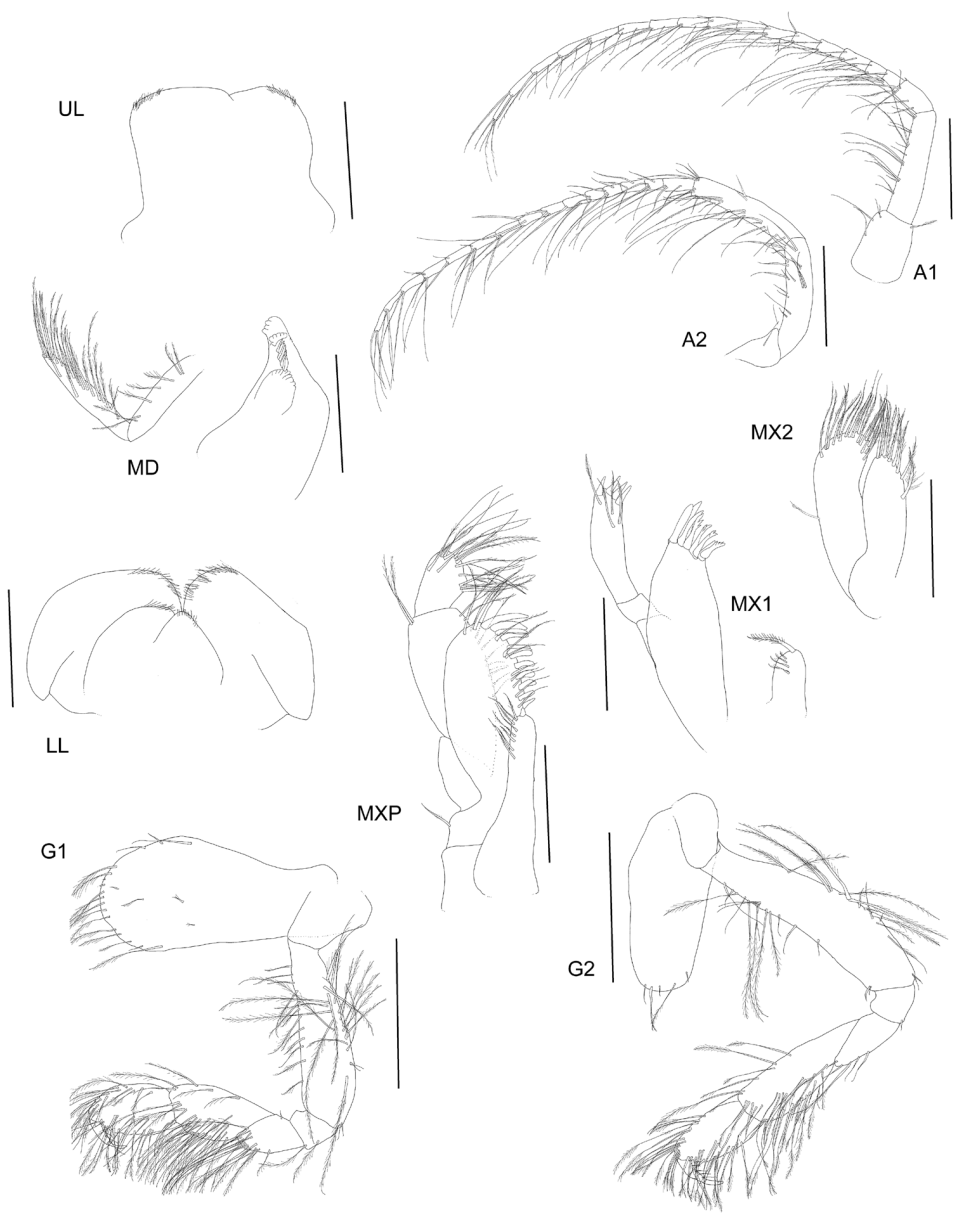
*Haploops dauvini* sp. n., holotype, female, 7 mm, ZMH K-47038, Norwegian Channel, 587.4–614.4 m. Antennae 1–2, gnathopods 1–2 scales represent 0.5 mm. Mouthparts scales represent 0.2 mm.

**Figure 7.**
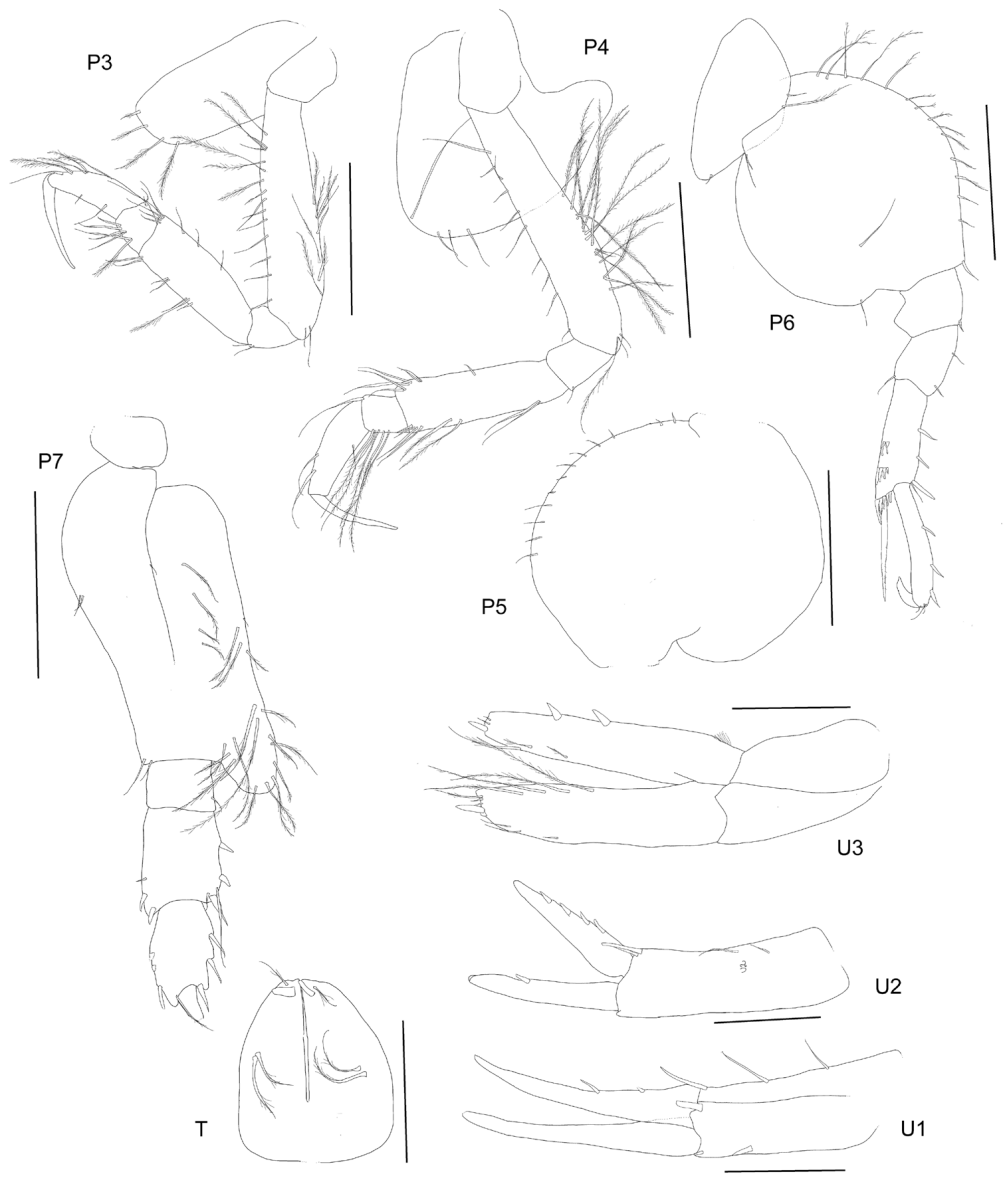
*Haploops dauvini* sp. n., holotype, female, 7 mm, ZMH K-47038, Norwegian Channel, 587.4–614.4 m. Pereopods 3–7 scales represent 0.5 mm. Uropods 1–3 and Telson scales represent 0.2 mm.

**Figure 8.**
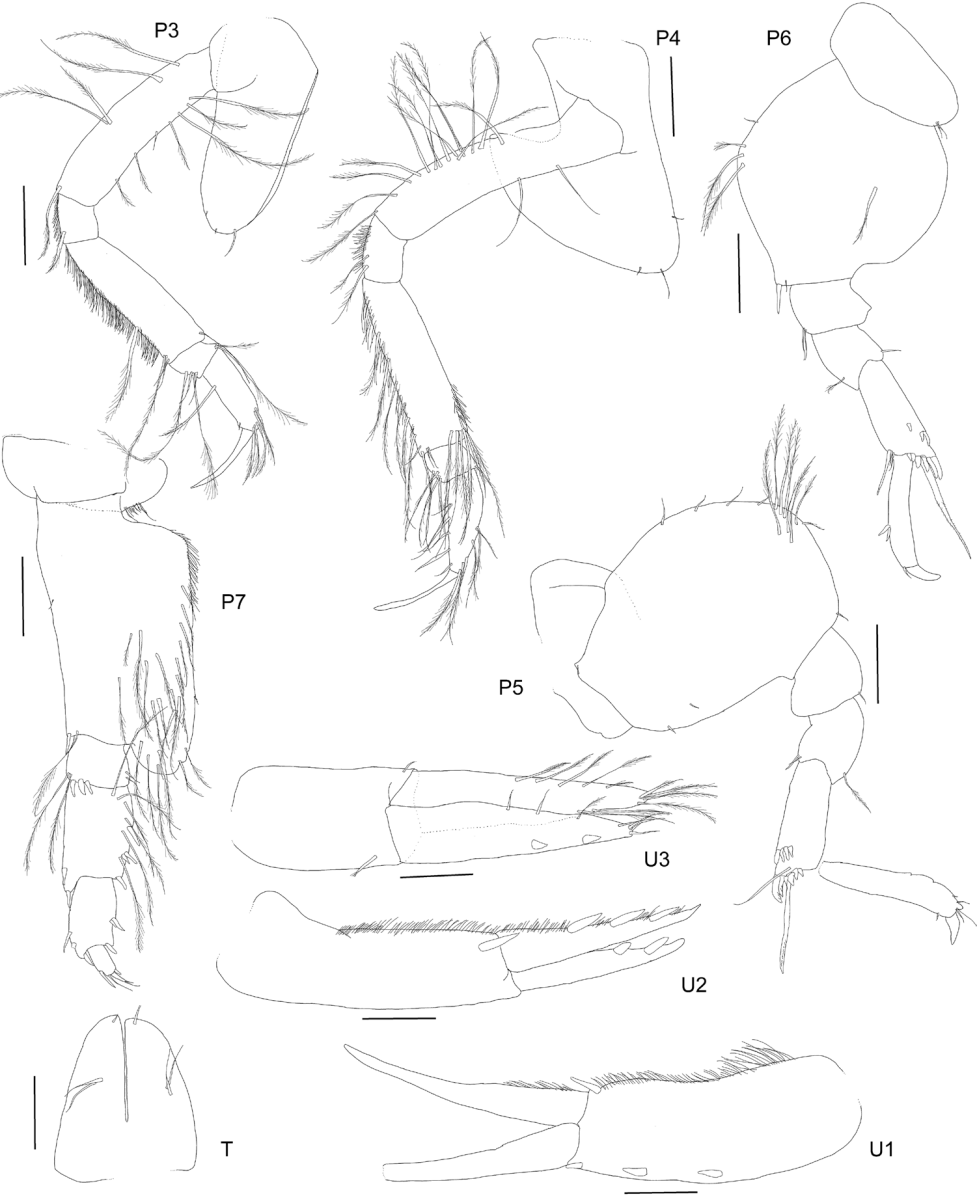
*Haploops kaimmalkai* sp. n. holotype, female, 6 mm, ZMH K-47057, Iceland Basin, 1384.8–1389 m. Whole animal, head and epimeron. Habitus scale represents 1 mm.

**Figure 9.**
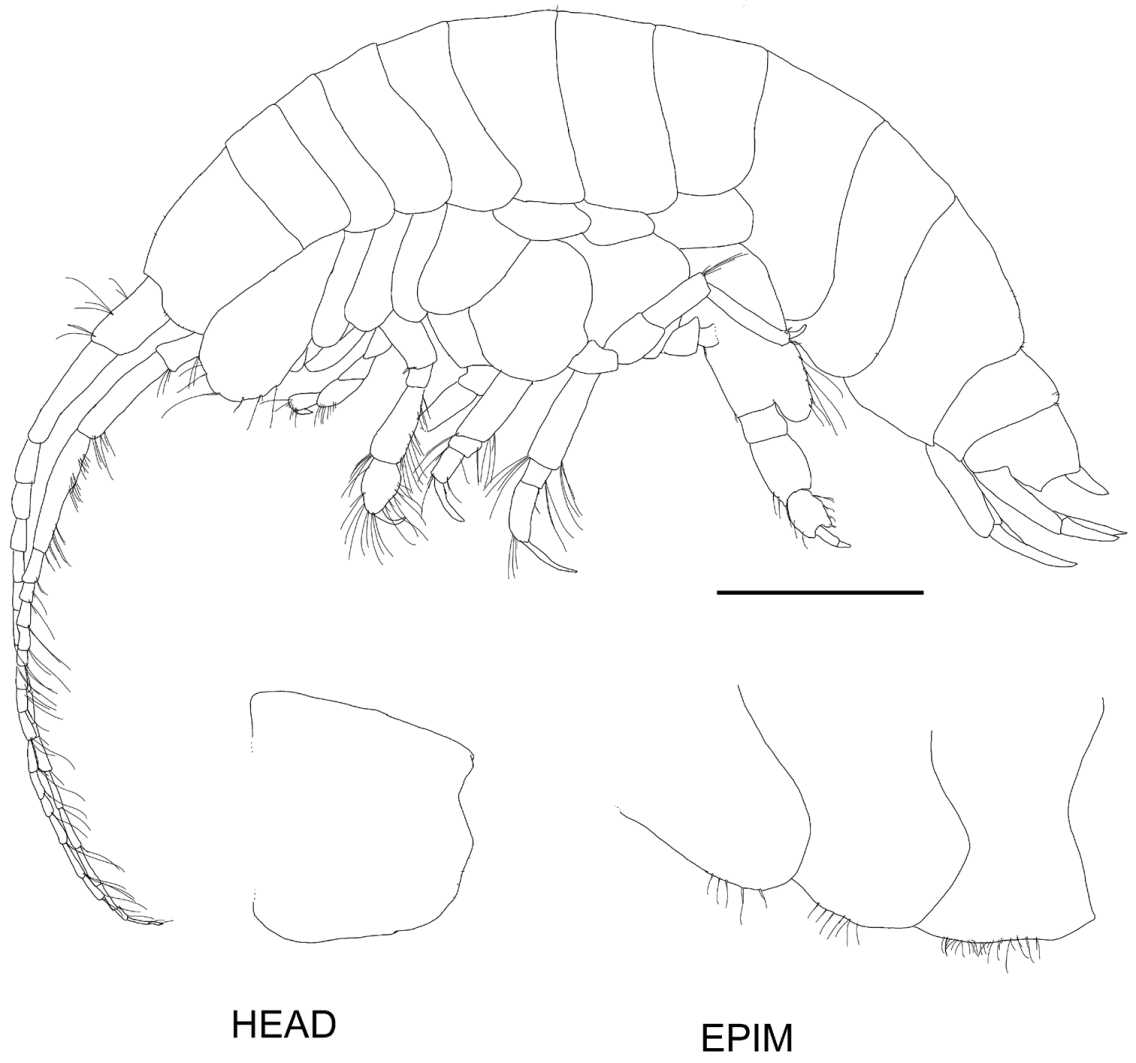
*Haploops kaimmalkai* sp. n. holotype, female, 6 mm, ZMH K-47057, Iceland Basin, 1384.8–1389 m. Scales represent 0.2 mm.

**Figure 10.**
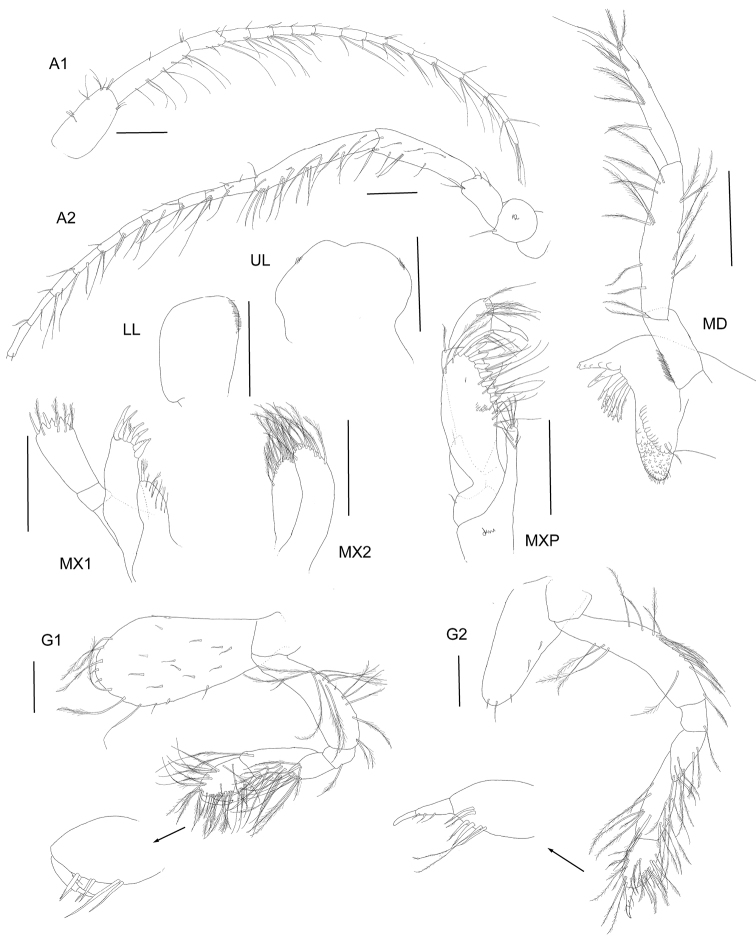
*Haploops kaimmalkai* sp. n. holotype, female, 6 mm, ZMH K-47057, Iceland Basin, 1384.8–1389 m. Pereopods 3–7 scales represent 0.2 mm. Uropods 1–3 and Telson scales represent 0.1 mm.

##### Remarks.

Of the blind species in *Haploops*, this species can be most closely allied to *Haploops abyssorum* Chevreux, 1908, *H. lodo* J.L. Barnard, 1961, *H. similis* Stephensen, 1925 and *H. kaimmalkai* (current work). The similarities between these species are due to the lack of eyes and a narrow pereopod 7 basis. The differences between the five species are shown in table 2.

**Table 2.** Character differences between five blind *Haploops* species.

Character	*H. dauvini*	*H. kaimmalkai*	*H. abyssorum*	*H. lodo*	*H. similis*
Head – anterior margin	straight	sinusoidal	straight	sinusoidal	straight
A1 - length	subequal to A2	subequal to A2	A1 shorter than A2, but longer than A2 peduncle	A1 shorter than A2, reaching to end of A2 peduncle	A1 shorter than A2 but longer than A2 peduncle
A1 art 1/art 2/art3	12.4/20/6.7	17.4/20/7.8	18/20/8	15.6/20/7.1	11.5/20/6.1
A2 art 4/art 5	art 4 = art 5	art 4 < art 5	art 4 < art 5	art 4 = art 5	art 4 = art 5
MD molar	medium and triturating	large and strongly triturating	?	not documented	Medium and triturating
MXP	outer plate reaching ¾ palp art 2 (in situ)	outer plate reaching equal topalp art 2 (in situ)	?	outer plate reaching to 1/3 palp art 3 (in situ)	Outer plate reaching ¾ palp art 2 (in situ)
MXP palp article 4	inserted slightly subterminally, longer than art 3, inner margin setose	inserted considerably subterminally, subequal to art 3, inner margin smooth	?	inserted slightly subterminally, longer than art 3, inner margin setose	Inserted considerably subterminally, subequal to art 3, inner margin smooth
Gnathopod 1Coxa	reaching to head anterior margin,ventrally smooth	reaching slightly past head anterior margin,ventrally slightly serrate	reaching beyond the head anterior margin, ventrally serrate	reaching beyond the head anterior margin, ventrally smooth	position to head anterior margin unknown, ventrally smooth
G1	carpus subequal in length to propodus	carpus longer than propodus	carpus slightly shorter than propodus	carpus longer than propodus	carpus longer than propodus
Coxa 2–3 ventral margin	long plumose setae	short simple setae	?	short simple setae	short simple setae
P3 ischium and merus	no strong marginal setal fringe, just clumps on both anterior and posterior margins of merus	strong posterior marginal setal fringe; anterior margin no setae	?	no strong marginal setal fringe; merus anterior margin without setae	No marginal setal fringe, posterior margin of merus with occasional plumose setae, anterior margin without setae
P4 coxa	broad	narrow	broad	broad	broad
P4 basis	plumose setae on both margins	plumose setae on posterior margin only	plumose setae on both margins	plumose setae on posterior margin only	plumose setae on posterior margin and sparsely on anterior margin
P4 distal articles posterior margin	fine setal fringe absent	fine setal fringe present	fine setal fringe absent	fine setal fringe absent	fine setal fringe absent
P4 dactylus/ propodus	dactylus < propodus	dactylus < propodus	dactylus > propodus	dactylus > propodus	dactylus < propodus
P5 basis	both margins broadly rounded	anterior margin broadly rounded, posterior margin sinusoidal	?	anterior margin narrowly rounded, posterior margin straight/truncated	Anterior margin weakly rounded, posterior margin weakly rounded
P5–6	propodus produced distally to form a lobe	propodus not produced distally, no lobe	?	propodus not produced distally, no lobe	propodus not produced distally, no lobe
P7 basis	concave margins, lobe deflected	straight margins, lobe not deflected	straight margins, lobe not deflected	concave margins, lobe deflected	Concave margins, lobe not deflected
P7 ischium	no anterodistal robust setae	three anterodistal robust setae	no anterodistal robust setae	five anterodistal robust setae	four anterodistal robust setae
P7 merus posterior margin	three robust setae, no plumose setae	four robust setae plus six long plumose setae	four robust setae, no plumose setae	three robust setae, no plumose setae	two robust setae, two distal long plumose setae
P7 merus/carpus/ propodus/dactylus	10.4/10/4.4/?	13.2/10/4.9/2.4	10.9/10/7.1/4.3	10.6/10/3.5/2.7	13/10/5/4
Epimeron 3: posteroventral corner	narrowly rounded, no tooth, sparse ventral setae	subquadrate, tiny tooth, ventral fringe of setae	subquadrate?	subquadrate, tiny tooth, no ventral setae	Subquadrate, no tooth, no ventral setae
Urosomite 1	weak rounded carina	weak rounded carina	carinate?	weak rounded carina	no carina
Uropod 1	peduncle shorter than rami	peduncle longer than rami	peduncle subequal to rami	peduncle slightly longer than rami	peduncle longer than rami
Uropod 1 rami length	rami subequal in length	rami subequal in length	rami subequal in length	inner ramus slightly shorter than outer	inner ramus shorter than outer
Uropod 1 inner rami	2 robust setae, no setal fringe	0 robust setae, setal fringe on proximal half	0 robust setae, no setal fringe	0 robust setae, slight setal fringe	1 robust seta (inner margin), no setal fringe
Uropod 2 rami length	rami subequal in length	outer ramus shorter than inner	outer ramus shorter than inner	outer ramus slightly longer / subequal to inner	outer ramus slightly shorter than inner
Uropod 2 inner ramus	4 robust setae, no setal fringe	3 robust setae, strong setal fringe	3 robust setae, no setal fringe	3 robust setae, no setal fringe	3 robust setae, no setal fringe
Telson	1 distal robust seta and 2 medial plumose setae per lobe	0 distal robust setae (just 1 slender seta) and 2 medial plumose per lobe	0 distal robust setae and 0 medial plumose setae per lobe	1 distal robust seta and 2 medial slender setae per lobe	1 distal robust seta and 0 medial setae per lobe

## Supplementary Material

XML Treatment for Byblisoides

XML Treatment for Byblisoides bellansantiniae

XML Treatment for Haploops

XML Treatment for Haploops dauvini
